# Metrology-Assisted Production in Agriculture and Forestry

**DOI:** 10.3390/s24237542

**Published:** 2024-11-26

**Authors:** H. R. Bogena, C. Brogi, C. Hübner, A. Panagopoulos

**Affiliations:** 1 Agrosphere Institute (IBG-3), Forschungszentrum Jülich GmbH, 52428 Jülich, Germany; 2 Department of Electrical Engineering, University of Applied Sciences Mannheim, Paul-Wittsack-Str. 10, 68163 Mannheim, Germany; 3 Soil and Water Resources Institute, Hellenic Agricultural Organization, Gorgopotamou Str., Sindos, 57400 Thessaloniki, Greece

According to the Food and Agriculture Organization of the United Nations, climate change will negatively affect food security and increase pressure on freshwater resources. Current scientific endeavors should thus provide reliable and robust information for management practices in agriculture and forestry that ensure a sustainable use of environmental resources and guarantee their resilience to the impacts of climate change. For instance, having spatiotemporal information on soil water content (SWC) and other environmental variables is key in most decision-making processes of farmers and growers, such as when a field can be driven on, when and how much irrigation should be applied, and when the use of fertilizers or pesticides is advisable or necessary. Furthermore, this information can assist farmers in determining the optimal harvest period and associated yield. The planning, execution, and effectiveness of such operations generally relies on extensive datasets obtained from in situ sensors, laboratory analysis, and drone- and remote sensing-based platforms. Farmers would thus benefit from an increased availability of real-time data and/or forecasts on the development of SWC, soil temperature, meteorological quantities, crop water requirements, and the availability of freshwater resources. To this end, modern agriculture and forestry are becoming more and more data-driven, and the adoption of sensor technology, data acquisition services, and advanced data processing and analysis capabilities is a key factor for the simultaneous increase in the sustainability and productivity of agricultural and forestry operations.

The purpose of this topical collection is to give an overview of the most recent advances made in the field of metrology-assisted production in agriculture and forestry and their applications in diverse areas. This collection features 15 articles, which are briefly introduced below.

Fragkos et al. [1] tested the performance of the TEROS 12 sensor for measuring SWC, electrical conductivity (σb), and temperature under laboratory conditions for different soils and conductivities. Six porous media and four solutions with increasing conductivity (0.28 to 10 dS/m) were tested. TEROS 12 showed lower permittivity values than Topp’s relationship, especially at high water content in sandy soils. The relationship between experimentally measured SWC and apparent dielectric permittivity was strongly linear but decreased with increasing σb. The multipoint-based calibration (CAL) provided the most accurate results.

Continuous monitoring of soil water content with capacitance sensors requires site-specific calibration, especially for clay-rich soils, as temperature effects on bound water are ignored. To this end, [2] developed a multi-point calibration for two clay-rich soils at temperatures from 10 to 40 °C and tested with GS3 and TEROS-12 sensors. Apparent dielectric bulk permittivity and SWC showed temperature-dependent relationships that were adjusted by a linear function. The temperature correction reduced the RMSE values to 0.007 to 0.033 cm^3^ cm^−3^, compared to 0.046 to 0.11 cm^3^ cm^−3^ at factory settings. The method was successfully validated at two locations.

SWC sensors are promising for climate-smart agriculture because they are easy to use and can take measurements at different depths. To this end, [3] evaluated three sensors (SoilVUE10, Drill&Drop, and SMT500) in terms of measurement accuracy, sensor-to-sensor variability, and temperature stability. Laboratory experiments in a temperature-controlled lysimeter showed that the Drill&Drop sensor had the highest temperature sensitivity (0.014 m^3^ m^−3^ per 10 °C), but the lowest sensor-to-sensor variability. In the field, the performance of all three sensor was similar (average RMSE ≈ 0.023 m^3^ m^−3^), with higher uncertainties at medium SWC. The combination of laboratory and field tests is well suited for the evaluation of SWC sensors.

Extreme weather events due to climate change are increasing, raising the demand for irrigation in agriculture, which is already the largest consumer of water. To promote efficient water use, [4] developed a novel sensor that measures soil water potential (SWP) rather than SWC. This sensor features two highly porous ceramic discs and a circuit board system utilizing time-domain transmission (TDT) to detect changes in the dielectric response based on water uptake. Field tests showed that the sensor’s signals correlate with soil water potential, regardless of soil composition, making it suitable for optimizing irrigation systems.

Becker et al. [5] tested a gamma-ray spectroscopy (GRS) method for the non-invasive continuous measurement of SWC at field level. To this end, a three-year field validation study in Nebraska, USA, investigated this in 27 measurement campaigns. The results showed that the current method for correcting biomass water content is suitable for corn and soybean, but the ratio of mass attenuation for soil and water needs to be adjusted. A calibration equation with two parameters is proposed to provide accurate SWC estimates. It is recommended to use 10 profiles and five calibration campaigns to achieve an accuracy of 0.035 g g^−1^.

The accuracy of SWC measured by Cosmic-Ray Neutrons Sensors (CRNSs) strongly depends on the Poisson-distributed count rate. Davies et al. [6] tested signal processing methods to improve the temporal accuracy of CRNS signals and to capture sub-daily changes in SWC. The moving average (MA), median filter (MF), Savitzky–Golay (SG) filter, and Kalman filter were analyzed for error reduction. Using synthetic data from four stations in Africa and Europe, the study found that smaller window sizes (12 h) for MA, MF, and SG captured rapid changes well, while longer windows were favorable for moderate SWC variations. The Kalman filter showed high robustness and captured sharp changes without optimal window size. Standard pre-filter corrections improved SWC measurements for all filters. This could significantly improve CRNS applications such as in detecting rain events and providing SWC data at the exact time of a satellite overpass.

Morris et al. [7] used CRNSs to measure SWC considering all sources of hydrogen, including variable plant biomass. Three fields in Nebraska were monitored for 5 and 13 years, respectively. Epithermal neutron counts, atmospheric variables, and point-scale SWC data were collected. In 2023, gravimetric SWC data were collected over the entire vegetation period. The N0 parameter was found to have a linear relationship with biomass water volume (BWE), suggesting a simple vegetation correction. The results were consistent with previous studies and provided new insights into the correction of CRNS measurements.

CRNSs offer potential for monitoring SWC in irrigated agriculture but presents specific challenges in small, irrigated fields. To investigate these in more detail, [8] deployed CRNSs in two apple orchards of about 1.2 ha in Greece. The CRNS measurements were compared with a dense sensor network. In 2021, the CRNSs only recorded the timing of irrigation, while an ad hoc calibration offered only limited improvements. In 2022, a correction based on neutron transport simulations reduced the RMSE from 0.052 to 0.031. This enabled more accurate monitoring of SWWC dynamics through irrigation and represents an advancement for CRNSs as a decision support system in irrigation management.

In recent years, wireless sensor network (WSN) technology has become increasingly important for SWC monitoring. In their review article, [9] describe the current status of WSN technology for distributed, near real-time measurement of SWC and its role in the validation and downscaling of satellite data, the validation of hydrological models, and to support agricultural management. Finally, perspectives for WSN measurements are highlighted, including better integration of real-time data with other information sources.

Zhang et al. [10] present the MSGV-YOLOv7 model for optimising the efficiency of pineapple harvesting robots. It uses MobileOne as the backbone and a thin neck to improve feature extraction and fusion, which increases the recognition rate. Results show that MSGV-YOLOv7 outperforms the original YOLOv7 with an increase in precision by 1.98%, recall rate by 1.35%, and mAP by 3.03%, with a recognition speed of 17.52 fps. Compared to Faster R-CNN and YOLOv5n, the mAP increased by 14.89% and 5.22%, respectively. The model shows potential for broad application to reduce time and costs in pineapple harvesting.

Accurate flower recognition is crucial for flower yield estimation. Zhao et al. [11] present the improved CR-YOLOv5s model, which integrates an attention mechanism to better recognize chrysanthemum buds and flowers in complex backgrounds. The coordinate attention mechanism improves the accuracy and robustness of the model. The results show an average accuracy of 93.9%, which is 4.5% better than normal YOLOv5s. This research supports the automatic harvesting and evaluation of flowers and provides a decision basis for yield estimation.

Accurate tree canopy detection is crucial for estimating yield in orchards. Cheng et al. [12] present an improved LA-dpv3+ model for cherry tree canopy detection from UAV image data that uses an attention mechanism to improve feature representation. The approach integrates this mechanism into the encoder stage of the DeepLabV3+ architecture, which increases the accuracy and robustness of the detection. The model is based on a lightweight DeepLabv3+ architecture with a MobileNetv2 backbone, which reduces computational costs. The accuracy of the model exceeded 89%, with a size of only 46.8 MB. The performance showed significant improvements in accuracy, F1 score, and intersection over union (IOU) by 5.44%, 3.39%, and 8.62%, respectively. The method has potential for future applications in automated orchard management.

A new satellite-based service, SENSE-GDD, provides continuous time series of Growing Degree Days (GDDs) with high spatial and temporal resolution. GDDs are calculated from MSG-SEVIRI data with an improvement in resolution from 4–5 km to 1 km. Keramitsoglou et al. [13] evaluated the performance of SENSE-GDD using temperature measurements in vineyards and apple orchards in Greece. The results show that SENSE-GDD provides reliable measurements during important phenological phases and supports cost-effective decisions in non-instrumented fields, promoting its application in agriculture.

The precise separation of rice grains is crucial for rice processing. Yu et al. [14] carried out simulations of the movement of rice grains in a clamped cylinder separator. The effects of factors such as cylinder rotation speed, angle of inclination and the inclination of the collecting chute were analyzed. The Kullback–Leibler divergence was used to evaluate the differences in the probability distributions of the escape angles of the grains. The aim is to determine the optimum parameters for the separator and to create a basis for the numerical design of grain cylinders.

Soil degradation and declining soil fertility are major challenges for sustainable agriculture in China. Promoting the use of conservation farming techniques is therefore crucial. Ren et al. [15] investigate how risk perception and social learning processes influence the adoption of these technologies. Using survey data from 1268 farmers in Shaanxi, Shanxi, and Ningxia provinces, an analysis with a binary probit model shows that risk perception positively influences the adoption of conservation farming techniques. Social learning reinforces this effect, with both practical learning and online learning playing an important role. The results emphasize that farmers’ risk perception should be taken into account when promoting these technologies and that the development of social learning channels is crucial.

We believe that the presented studies will contribute to the advancement of metrology-assisted production in agriculture and forestry.

## References

[1] Fragkos A., Loukatos D., Kargas G., Arvanitis K.G. (2024). Response of the TEROS 12 Soil Moisture Sensor under Different Soils and Variable Electrical Conductivity. Sensors.

[2] Nasta P., Coccia F., Lazzaro U., Bogena H.R., Huisman J.A., Sica B., Mazzitelli C., Vereecken H., Romano N. (2024). Temperature-corrected calibration of GS3 and TEROS-12 soil water content sensors. Sensors.

[3] Nieberding F., Huisman J.A., Huebner C., Schilling B., Weuthen A., Bogena H.R. (2023). Evaluation of Three Soil Moisture Profile Sensors Using Laboratory and Field Experiments. Sensors.

[4] Menne D., Hübner C., Trebbels D., Willenbacher N. (2022). Robust soil water potential sensor to optimize irrigation in agriculture. Sensors.

[5] Becker S.M., Franz T.E., Morris T.C., Mullins B. (2024). Field Testing of Gamma-Spectroscopy Method for Soil Water Content Estimation in an Agricultural Field. Sensors.

[6] Davies P., Baatz R., Bogena H.R., Quansah E., Amekudzi L.K. (2022). Optimal temporal filtering of the cosmic-ray neutron signal to reduce soil moisture uncertainty. Sensors.

[7] Morris T.C., Franz T.E., Becker S.M., Suyker A.E. (2024). Effect of Biomass Water Dynamics in Cosmic-Ray Neutron Sensor Observations: A Long-Term Analysis of Maize–Soybean Rotation in Nebraska. Sensors.

[8] Brogi C., Pisinaras V., Köhli M., Dombrowski O., Hendricks Franssen H.-J., Babakos K., Chatzi A., Panagopoulos A., Bogena H.R. (2023). Monitoring irrigation in small orchards with cosmic-ray neutron sensors. Sensors.

[9] Bogena H.R., Weuthen A., Huisman J.A. (2022). Recent developments in wireless soil moisture sensing to support scientific research and agricultural management. Sensors.

[10] Zhang R., Huang Z., Zhang Y., Xue Z., Li X. (2023). MSGV-YOLOv7: A Lightweight Pineapple Detection Method. Agriculture.

[11] Zhao W., Wu D., Zheng X. (2023). Detection of chrysanthemums inflorescence based on improved CR-YOLOv5s algorithm. Sensors.

[12] Cheng Z., Cheng Y., Li M., Dong X., Gong S., Min X. (2023). Detection of cherry tree crown based on improved LA-dpv3+ algorithm. Forests.

[13] Keramitsoglou I., Sismanidis P., Sykioti O., Pisinaras V., Tsakmakis I., Panagopoulos A., Argyriou A., Kiranoudis C.T. (2023). SENSE-GDD: A Satellite-Derived Temperature Monitoring Service to Provide Growing Degree Days. Agriculture.

[14] Yu X., Jiang X., Gu H., Shen F. (2022). DEM Study of the Motion Characteristics of Rice Particles in the Indented Cylinder Separator. Sensors.

[15] Ren Y., Feng H., Gao T. (2023). Risk cognition, social learning, and farmers’ adoption of conservation agriculture technology. Agriculture.

